# Piezo1-pannexin-1-P2X_3_ axis in odontoblasts and neurons mediates sensory transduction in dentinal sensitivity

**DOI:** 10.3389/fphys.2022.891759

**Published:** 2022-12-14

**Authors:** Sadao Ohyama, Takehito Ouchi, Maki Kimura, Ryuya Kurashima, Keiko Yasumatsu, Daisuke Nishida, Suzuro Hitomi, Sobhan Ubaidus, Hidetaka Kuroda, Shinichirou Ito, Masayuki Takano, Kentaro Ono, Toshihide Mizoguchi, Akira Katakura, Yoshiyuki Shibukawa

**Affiliations:** ^1^ Department of Physiology, Tokyo Dental College, Tokyo, Japan; ^2^ Oral Surgery, Tokyo Metropolitan Cancer and Infectious Diseases Center, Komagome Hospital, Tokyo, Japan; ^3^ Tokyo Dental Junior College, Tokyo, Japan; ^4^ Oral Health Science Center, Tokyo Dental College, Tokyo, Japan; ^5^ Department of Physiology, Nihon University School of Dentistry, Tokyo, Japan; ^6^ Division of Physiology, Kyushu Dental University, Fukuoka, Japan; ^7^ Department of Dental Anesthesiology, Kanagawa Dental University, Yokosuka, Japan; ^8^ Department of Oral and Maxillofacial Surgery, Tokyo Dental College, Tokyo, Japan; ^9^ Department of Oral Pathological Science and Surgery, Tokyo Dental College, Tokyo, Japan

**Keywords:** dental pain, dentin hypersensitivity, odontoblasts, pannexin channels, piezo channels, P2X receptors, tooth pain

## Abstract

According to the “hydrodynamic theory,” dentinal pain or sensitivity is caused by dentinal fluid movement following the application of various stimuli to the dentin surface. Recent convergent evidence *in Vitro* has shown that plasma membrane deformation, mimicking dentinal fluid movement, activates mechanosensitive transient receptor potential (TRP)/Piezo channels in odontoblasts, with the Ca^2+^ signal eliciting the release of ATP from pannexin-1 (PANX-1). The released ATP activates the P2X_3_ receptor, which generates and propagates action potentials in the intradental Aδ afferent neurons. Thus, odontoblasts act as sensory receptor cells, and odontoblast-neuron signal communication established by the TRP/Piezo channel-PANX-1-P2X_3_ receptor complex may describe the mechanism of the sensory transduction sequence for dentinal sensitivity. To determine whether odontoblast-neuron communication and odontoblasts acting as sensory receptors are essential for generating dentinal pain, we evaluated nociceptive scores by analyzing behaviors evoked by dentinal sensitivity in conscious Wistar rats and Cre-mediated transgenic mouse models. In the dentin-exposed group, treatment with a bonding agent on the dentin surface, as well as systemic administration of A-317491 (P2X_3_ receptor antagonist), mefloquine and ^10^PANX (non-selective and selective PANX-1 antagonists), GsMTx-4 (selective Piezo1 channel antagonist), and HC-030031 (selective TRPA1 channel antagonist), but not HC-070 (selective TRPC5 channel antagonist), significantly reduced nociceptive scores following cold water (0.1 ml) stimulation of the exposed dentin surface of the incisors compared to the scores of rats without local or systemic treatment. When we applied cold water stimulation to the exposed dentin surface of the lower first molar, nociceptive scores in the rats with systemic administration of A-317491, ^10^PANX, and GsMTx-4 were significantly reduced compared to those in the rats without systemic treatment. Dentin-exposed mice, with somatic odontoblast-specific depletion, also showed significant reduction in the nociceptive scores compared to those of Cre-mediated transgenic mice, which did not show any type of cell deletion, including odontoblasts. In the odontoblast-eliminated mice, P2X_3_ receptor-positive A-neurons were morphologically intact. These results indicate that neurotransmission between odontoblasts and neurons mediated by the Piezo1/TRPA1-pannexin-1-P2X_3_ receptor axis is necessary for the development of dentinal pain. In addition, odontoblasts are necessary for sensory transduction to generate dentinal sensitivity as mechanosensory receptor cells.

## Introduction

The removal of enamel exposes dentin and renders it extremely sensitive to thermal (such as cold), mechanical (such as scraping, cutting, and drilling dentin), chemical (such as low pH solutions), and osmotic (such as hypertonic solutions) stimuli. This produces unendurable pain in the tooth known as “dentinal pain,” often resulting in “dentin hypersensitivity.” Dentin hypersensitivity is an important oral health problem worldwide, with a high prevalence (8–57% in the general population) (Canadian Advisory Board on Dentin Hypersensitivity, 2003), which can cause psychological distress and reduce overall quality of life (de Oliveira and Nadanovsky, 2006; Luo et al., 2007). However, the mechanisms underlying its occurrence are not well understood.

In 1900, Gysi first presented the “hydrodynamic theory” (Gysi, 1900; Bränström, 1963) to explain dentin sensitivity. In the 20th century, the hydrodynamic theory was widely accepted as the primary mechanism underlying the occurrence of dentinal sensitivity (Andrew and Matthews 2000; Charoenlarp et al., 2007). Dentin is permeated by dentinal tubules that contain dentinal fluid. These tubules act as a hydraulic link between dentin at the enamel-dentin junction (EDJ) or exposed dentin surface and at the dental pulp end of the tubules, where cellular components exist. The axon innervating the dental pulp forms a nerve plexus at the periphery of the dental pulp beneath the layer of odontoblasts, which primarily function as dentin-forming cells (Sessle, 1987). Changes in dentinal fluid volume following thermal, mechanical, chemical, and osmotic stimuli applied to the dentin surface induce outward movement of the dentinal fluid (i.e., movement from the pulpal side to the dentin surface), altering the hydrodynamic force inside the dentinal tubules (Lin et al., 2011). Electrophysiological recordings have shown that any stimulus that causes fluid flow in the dentinal tubules results in an increased discharge rate in the associated intradental afferent nerve fibers (Andrew and Matthews, 2000; Charoenlarp et al., 2007; Vongsavan and Matthews, 2007). This explains why diverse stimuli initiate a similar pattern of painful responses in dentinal sensitivity. Additionally, fluid flow in the dentinal tubules following various stimuli is sufficiently rapid to induce painful responses in the dentin, leading to mechanical effects on the tubules at the pulp/dentin border where nerve endings and odontoblasts are located. This also indicates that the actual stimulus applied to the “sensory receptor” of dentin is mechanical.

Recently, we proposed a model (the “odontoblast hydrodynamic receptor theory/model”; Shibukawa et al., 2015) which suggests that intercellular odontoblast-neuron signal transduction explains the sensory generation mechanism of dentinal pain. In this model, mechanical stimulation of odontoblasts resulting from deformation of the odontoblastic membrane by dentinal fluid movement (*via* hydrodynamic force inside dentinal tubules) induced transmembrane Ca^2+^ influx *via* activation of transient receptor potential (TRP)-vanilloid subfamily member-1, -2, and -4 (TRPV1, TRPV2, TRPV4); TRP-ankyrin subfamily member-1 (TRPA1) (Shibukawa et al., 2015); and Piezo1 channels (Sato et al., 2018; Matsunaga et al., 2021), all of which show mechanosensitivity. Activated TRP/Piezo channels act as mechanosensors in the plasma membrane to mediate the release of neurotransmitters, ATP, through pannexin-1 channels (PANX-1) (Velasquez and Eugenin, 2014) and establish intercellular odontoblast-neuron signal communication. ATP released from stimulated odontoblasts activates P2X_3_ receptors in trigeminal ganglion (TG) neurons, which innervate the dental pulp as sensory afferent neurons (Sato et al., 2015, 2018; Shibukawa et al., 2015). Indeed, the activation of P2X_3_ receptors upon ATP release from mechanically stimulated odontoblasts generates and propagates an action potential in the Aδ afferents of TG neurons (Sato et al., 2018). These convergent observations strongly support the idea that intercellular odontoblast-neuron signal transduction best describes the sensory transduction and generation mechanism of dentinal pain/sensitivity. However, whether the model is applicable at the organism level and whether odontoblasts are necessary to evoke dentinal sensitivity remain unclear.

In this study, we aimed to investigate whether mechanosensitive TRP/Piezo1 channels and PANX-1 in odontoblasts, and P2X_3_ receptors in TG neurons, as well as odontoblasts themselves are necessary to dentinal sensitivity *in vivo*. We analyzed pain behaviors induced by dentinal sensitivity to cold stimuli applied to the exposed dentin surface in rats and mice. We also evaluated the effects of pharmacological agents, antagonists for Piezo1, PANX-1, P2X_3_ receptor, TRP canonical subfamily member 5 (TRPC5), and TRPA1 channels on the nociceptive behavior of rats. Behaviors were also examined in genetically modified mice, allowing for efficient depletion of odontoblasts *in vivo* following somatic Cre-mediated gene recombination.

## Materials and methods

### Ethical approval

The animals used in this study [Wistar rats (*n* = 121, both sexes, 8–15 weeks old, weight 170–325 g) and mice (*n* = 16, all male, 8 weeks old, weighing 20–30 g)] were housed in transparent cages with wood chips under specific pathogen-free conditions and maintained on a light-dark cycle (12:12) in a temperature- and humidity-controlled room (21–23°C and 40–60%, respectively). Food pellets and water were provided *ad libitum*. All animals were treated in accordance with the Guiding Principles for the Care and Use of Animals in the field of physiological sciences, approved by the Council of the Physiological Society of Japan and the American Physiological Society. All animal experiments conducted in this study followed the Animal Research: Reporting of *In Vivo* Experiments guidelines; the guidelines of the National Institutes of Health, United States regarding the care and use of animals for experimental procedures; and the United Kingdom Animals (Scientific Procedures) Act, 1986. All experiments were approved by the Ethics Committee of our institute (Experimental Animal Plan approval number: 190301, 200301, 210301, 220301). All efforts were made to minimize animal suffering. All animals used in this study were healthy and presented no complications during the experimental period.

### Preparation of mice with odontoblast-specific depletion using somatic Cre-mediated gene recombination

Mice with odontoblast-specific depletion *via* somatic Cre-mediated gene recombination were obtained using a previously described method (Zhao et al., 2021). We prepared mice expressing a DNA recombinant enzyme (Cre) under the control of a gene in the 2.3-kb region of the collagen-1 promoter (referred to as “Col1 (2.3)-Cre mice” [B6. FVB-Tg (Col1a1-cre)1 Kry mice (RBRC05603); RIKEN BRC, Ibaraki, Japan]) (Dacquin et al., 2002). We also used mice carrying a gene for the flox-stop-flox-diphtheria toxin receptor, in which the diphtheria toxin (DT) receptor (DTR) is induced in a Cre expression-dependent manner (referred to as “DTR-mice” [C57BL/6-Gt (ROSA) 26Sortm1 (HBEGF) Awai/J (JAX007900), Jackson Laboratory, Bar Harbor, ME, United States]). To prepare mice exhibiting odontoblast-specific depletion following somatic Cre-mediated gene recombination and to generate mice carrying both genes, we crossed Col1 (2.3)-Cre and DTR-mice (referred as “Col1 (2.3)-Cre/DTR-mice”). In Col1 (2.3)-Cre/DTR-mice, odontoblasts specifically expressed DTR; thus, we could remove and deplete only odontoblasts at any time by administering DT (Zhao et al., 2021). For DT-mediated cell deficiency, 250 ng DT (Sigma-Aldrich, St. Louis, MO, United States) was injected intraperitoneally every 24 h for 1 week. To evaluate the nociceptive scores induced by dentinal pain (see below), we used Col1 (2.3)-Cre/DTR-mice (*n* = 8, all male, 8 weeks old, 20–28 g) 24 h after the final DT administration. Before and after a series of nociceptive score measurements, we dissected and collected incisors from Col1 (2.3)-Cre/DTR-mice and DTR-mice under anesthesia and observed tissue structure of the dentin-pulp border using hematoxylin-eosin staining and immunostaining (see below). We used DTR-mice as controls (*n* = 8, all male, 8 weeks old, weighing 20–30 g). DTR-mice showed no cell deletions, including odontoblasts. As another control, we administered DT to DTR-mice in a manner similar to that of Col1 (2.3)-Cre/DTR-mice and evaluated nociceptive scores induced by dentinal pain.

### Histology

We dissected and collected the incisors from DT-administered DTR-mice and Col1 (2.3)-Cre/DTR-mice, and the mandibles, including incisors and molars, from rats. The tissues were fixed at 4°C for 24 h in 0.1 M phosphate-buffered saline (PBS) containing 4% paraformaldehyde. The specimens were decalcified by storage in Morse’s solution at 4°C for 1 week. The decalcified tissues were dehydrated with ethanol and embedded in paraffin, and 5-µm thick slices were cut using a microtome (SM 2000R; Leica, Wetzlar, Germany). The slices from DT-administered DTR-mice and Col1 (2.3)-Cre/DTR-mice were then stained with hematoxylin and eosin and mounted on glass slides that were enclosed using an encapsulant (FX00100, Matsunami, Osaka, Japan). The slides were observed under a microscope (UPM Axiophoto2, Carl Zeiss, Tokyo, Japan), and images were extracted using Photoshop and Illustrator (Adobe, San Jose, CA).

### Immunostaining

After performing a series of measurements of nociceptive scores (both mice had been administered DT as described in the text), the incisors were also dissected and collected from DTR- and Col1 (2.3)-Cre/DTR-mice. In addition, mandibles form rats were also dissected and collected. The specimens were fixed, decalcified, dehydrated, and embedded in paraffin, and 5-µm-thick slices were prepared (see above). To completely remove paraffin from the sections, xylene was applied three times. Each treatment was performed for 10 min. After xylene treatment, the sections were placed in ethanol to remove the xylene. The ethanol concentration was gradually reduced from 100% to 95%, and then to 70%. Each section was placed in an ethanol bath for 10 min. After ethanol treatment, the sections were washed three times with PBS. For immunofluorescence analysis, the samples were incubated with blocking buffer (Nacalai Tesque, Kyoto, Japan) at room temperature for 15 min. To evaluate the protein expression patterns in the odontoblast layer and cell-rich zone in mouse incisors, we used the following primary antibodies overnight at 4°C: rabbit polyclonal anti-dentin sialophosphoprotein (DSPP; bs-8557R; 1:200; Bioss Antibodies Inc, Woburn, MA, United States) and mouse monoclonal anti-nestin (sc-23927 (10c2); 1:200; Santa Cruz Biotechnology, Dallas, Texas, United States). The secondary antibodies used were Alexa Fluor^®^ 568 donkey anti-rabbit (#A10042; Thermo Fisher Scientific, Tokyo, Japan) and Alexa Fluor^®^ 647 donkey anti-mouse (#A31571; Thermo Fisher Scientific). We also evaluated the expression patterns of A-neuron marker protein, anti-neurofilament heavy chain (NF-H), and ionotropic ATP receptor subtype, P2X_3_ receptors, in the dentin-pulp border of the incisors from DT-administered DTR-mice and Col1 (2.3)-Cre/DTR-mice, as well as the lower incisors and molars of rats. We used the following primary antibodies overnight at 4°C: mouse monoclonal anti-neurofilament heavy chain (NF-H; sc-32729 (RNF402); 1:100; Santa Cruz Biotechnology), and rabbit polyclonal anti-P2X_3_ receptors (ab10269; 1:100; Abcam, Cambridge, United Kingdom). The secondary antibodies used were Alexa Fluor^®^ 568 donkey anti-mouse (#A10037) and Alexa Fluor^®^ 488 donkey anti-rabbit (#A21206; Thermo Fisher Scientific). The secondary antibodies were applied for 1 h at room temperature in the dark. The stained samples were mounted in mounting medium with 4,6-diamidino-2-phenylindole (ab104139; Abcam). Immunofluorescence was observed using a fluorescence microscope (BZ-X710; Keyence, Osaka, Japan).

### Dentinal exposure preparation to induce dentinal sensitivity in rats and mice

**FIGURE 1:**
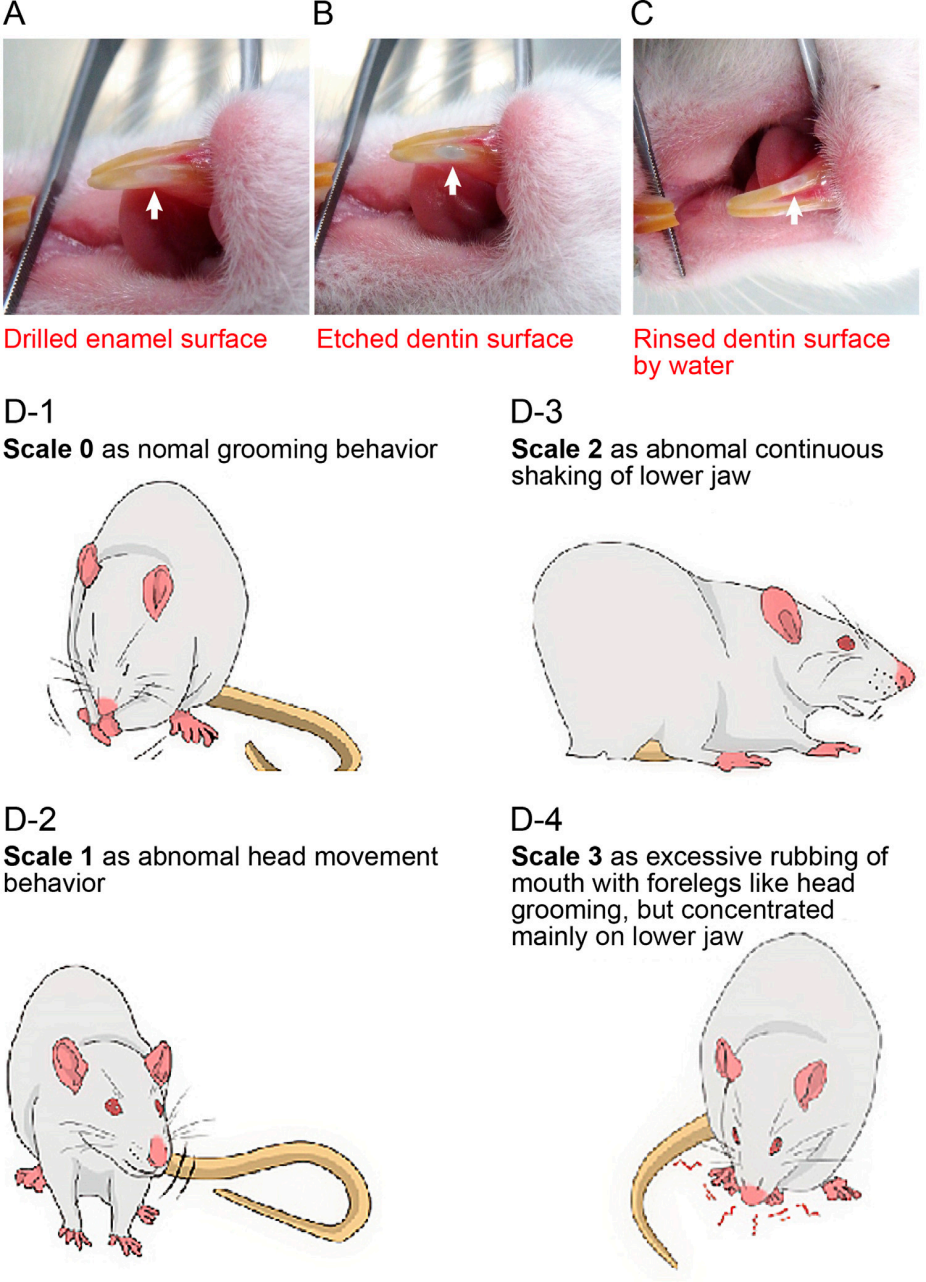
Preparation procedure for dentin exposure in mandibular incisors and evaluation of nociceptive scales **(A–C)** Photographs showing the preparation procedures to expose dentin of the incisors. We drilled a 1-mm-wide cavity in the enamel of the lower incisor to expose the dentin surface at the 1-mm rostral end of the gingival margin **(A)**. For molars, we drilled a 1-mm diameter in the enamel to expose the dentin surface, but not the pulp chamber, at the center of the occlusal surface of the right lower first molar. The exposed dentin surface was then etched to remove the smear layer **(B)**. The etched surface was rinsed with water to wash off the acid agent **(C)**. **(D)** Rats and mice were allowed to move freely in a clear acrylic gauge (45 cm × 30 cm × 30 cm) with a mirror to acclimatize them to the environment. Nociceptive behavior was measured for 180 s after stimulation and classified on a nociceptive scale from 0 to 3. A scale of 0 was defined as normal behavior, such as grooming **(D-1)**. A scale of 1 was chosen when rats or mice showed abnormal head movements or mild head shaking **(D-2)**. A scale of 2 indicates abnormal continuous shaking of the lower jaw **(D-3)**. A scale of 3 was chosen when they showed excessive rubbing of the mouth with foreleg movements, such as head grooming, but concentrated mainly in the lower jaw **(D-4)**. From these scales, we calculated the nociceptive score using the following formula:Nociceptive score = [(1 × Tscale 1)] + [2 × Tscale 2] + [(3 × Tscale 3])/180 (Equation 1). where Tscale (*n*) indicates the total time (s) exhibiting nociceptive behavior for each scale (*n*) during a 180-s recording period (Chidiac et al., 2002).

Wistar rats, DTR-mice, and Col1 (2.3)-Cre/DTR-mice were anesthetized with 3% isoflurane (FUJIFILM Wako Pure Chemical Co., Osaka, Japan). For the lower incisors, a 1-mm wide cavity was drilled 1-mm coronal to the gingival margin in the enamel to expose the dentin (Figures 1A–C) (dental motor from VIVA MATE G5 Nakanishi Inc, Kanuma, Japan; low-speed handpiece Torqtech from J. Morita Co., Suita, Japan; and dental diamond bur from Syofu Inc, Kyoto, Japan). For the right first molars of rats, a 1 mm diameter cavity was drilled at the center of the occlusal surface in the enamel to expose the dentin (not shown) (dental motor from VIVA MATE G5; low-speed handpiece Torqtech from J. Morita Co.; dental diamond bur, Syofu Inc.). The exposed dentin was etched (Sun Medical Company, Ltd., Moriyama, Japan) to induce dentinal pain, followed by cold stimulation of the dentin. Measurements of pain behavior elicited by dentinal sensitivity were made after full recovery from anesthesia (assessed by the present of spontaneous movement without wobbling, jaw tone, and withdrawal reflex) followed by initiating habituation period.

### Evaluation of nociceptive scores by measuring pain behavior elicited by dentinal sensitivity

**FIGURE 2:**
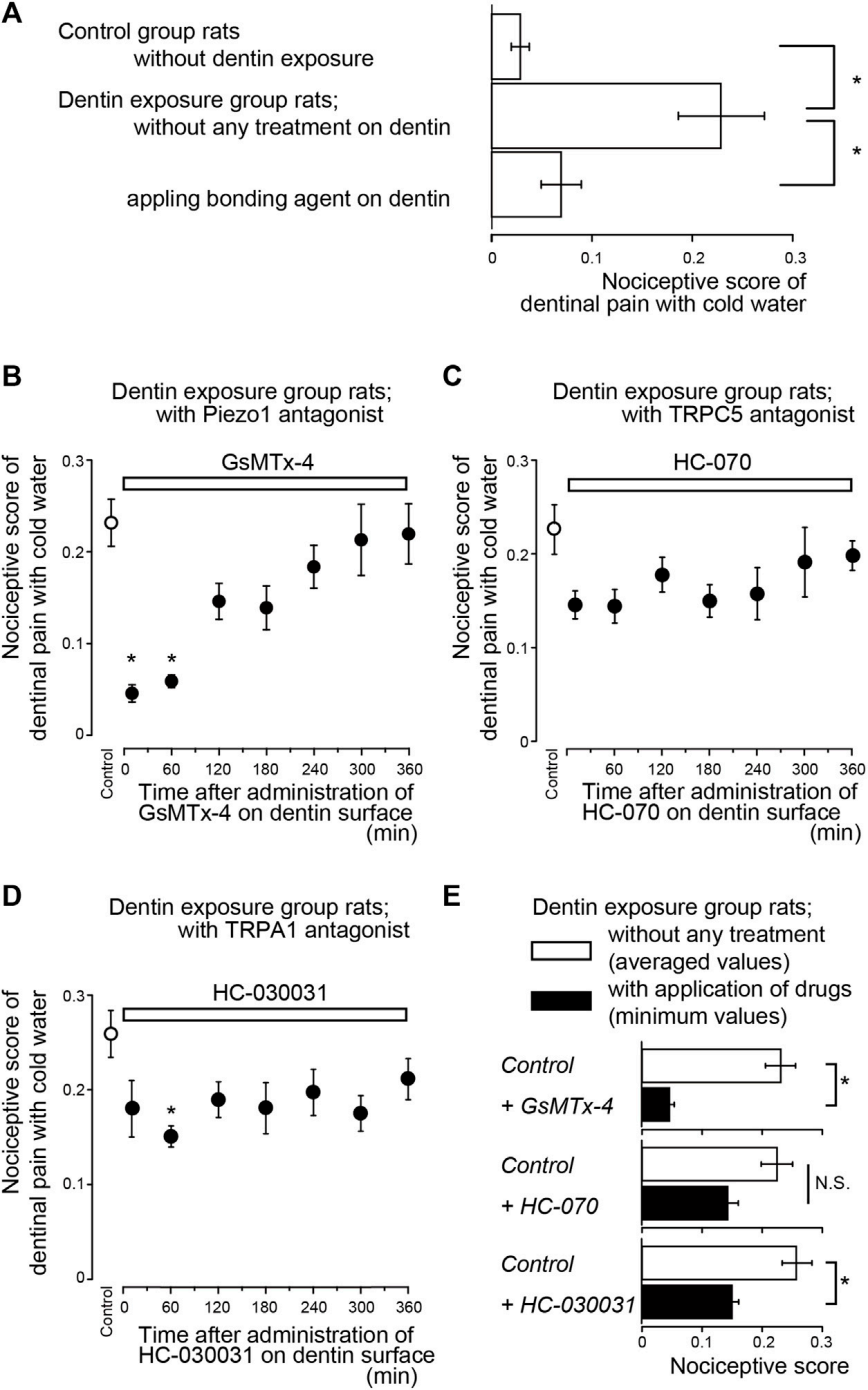
Time-dependence of pharmacological effects of Piezo1 (GsMTx-4), TRPC5 (HC-070), and TRPA1 (HC-030031) channel antagonists, as well as bonding agent, on dentinal sensitivity **(A)** Bar graph shows values of the nociceptive scores following cold water (0.1 ml, 4.0–7.0°C) stimulation of the dentin surface in rats in the control group in which the enamel surface was etched (upper column, *n* = 8), dentin-exposed rats without any treatment of the dentin surface (middle column, *n* = 10), and dentin-exposed rats treated with a bonding agent applied to the dentin surface (lower column, *n* = 8). Each bar denotes the mean ± SE of the number of experiments. Significant differences between columns (shown by solid lines) are indicated by asterisks: *p < 0.05 **(B–D)** Time-dependent effects of GsMTx-4 (B; *n* = 7, 0.8 nmol/g (weight) i.v.), HC-070 (C; *n* = 8, 0.008 nmol/g (weight), i.v.), and HC-030031 (D; *n* = 8, 8 nmol/g (weight), i.v.) (upper white boxes) on nociceptive scores following cold water stimulation. Prior to the systemic administration of GsMTx-4, HC-070, or HC-030031 to the dentin-exposed rats, we obtained nociceptive scores as control values (control; white circles). Several minutes after the measurements of the control value in the nociceptive scores, GsMTx-4 **(B)**, HC-070 **(C)**, or HC-030031 **(D)** were administered to the rats. The cold water stimuli-induced nociceptive scores were measured repeatedly at 10, 60, 120, 180, 240, 300, and 360 min later each (black circles in B to D; see also Materials and Methods). Each point denotes the mean ± SE of the number of experiments. Significant differences in the scores compared to control values are denoted by asterisks: *p < 0.05. **(E)** Summary bar graphs showing mean values of nociceptive scores in the dentin-exposed group of rats without any treatment (control; white columns), and their minimum values (during the entire length of administration) in dentin-exposed rats subjected to administration of GsMTx-4 (*n* = 7), HC-070 (*n* = 8), and HC-030031 (*n* = 8) (black columns). Each bar represents mean ± SE of the number of experiments. Significant differences between columns (shown by solid lines) are denoted by asterisks: *p < 0.05. N.S.; not significant.

**FIGURE 3:**
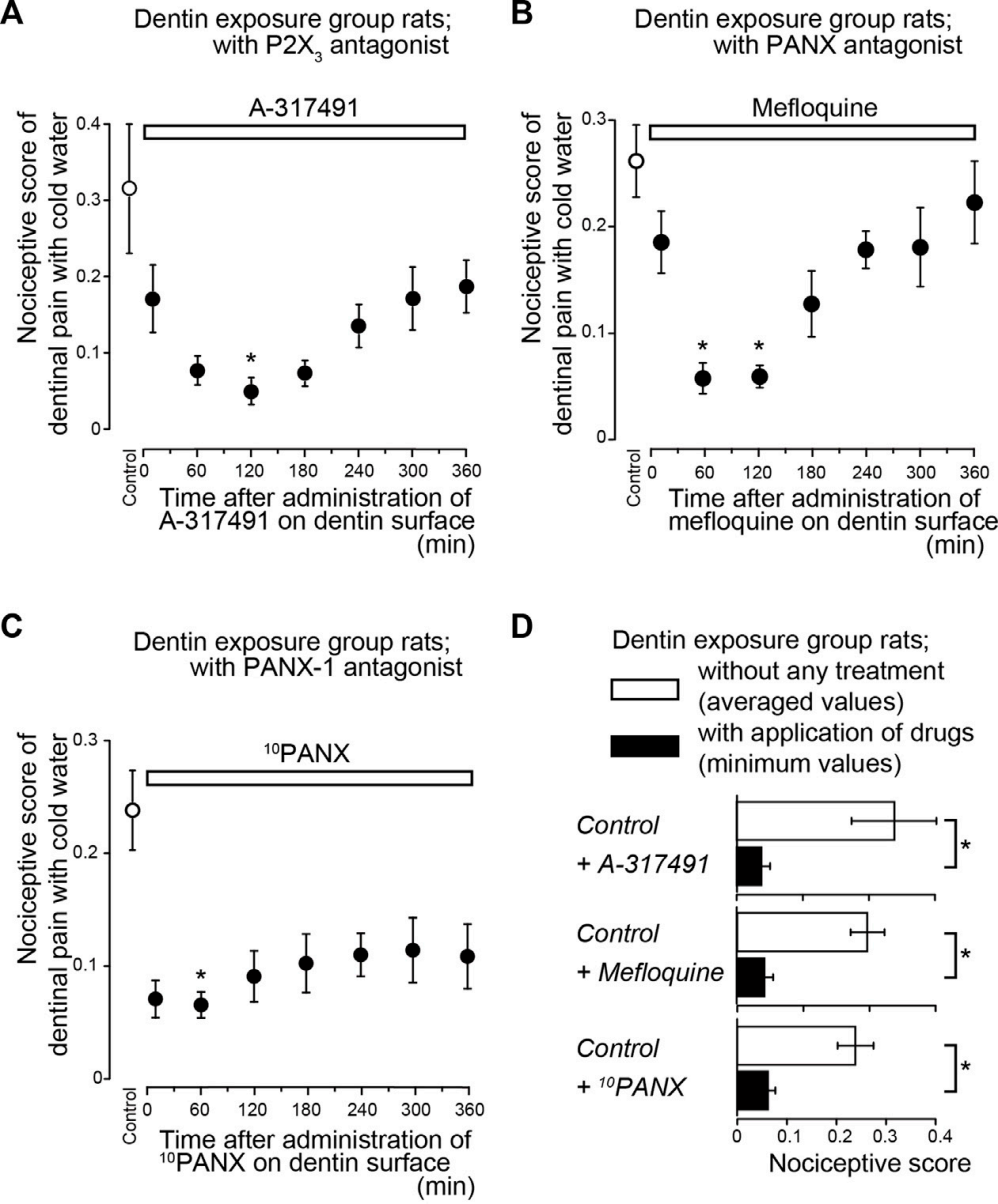
Time-dependence of pharmacological effects of P2X_3_ receptor (A-317491) and PANX (mefloquine and ^10^PANX) antagonist on dentinal sensitivity **(A–C)** Time-dependent effects of A-317491 (A; *n* = 6, 0.3 nmol/g (weight) i.v.), mefloquine (B; *n* = 7, 0.03 mg/g (weight) p.o.), and ^10^PANX (C; *n* = 8, 2.58 nmol/g (weight) i.v.) (upper white boxes) on nociceptive scores following cold water stimulation. Prior to the systemic administration of A-317491, mefloquine, or ^10^PANX to the dentin-exposed rats, nociceptive scores were determined as the control values (white circles). Several minutes after these measurements, A-317491 **(A)**, mefloquine **(B)**, or ^10^PANX **(C)** was administered to the rats. The cold water stimuli-induced nociceptive scores were measured repeatedly at 10, 60, 120, 180, 240, 300, and 360 min later each (black circles in A to C; see also Materials and Methods). Each point denotes the mean ± SE of the number of experiments. Significant differences in the scores compared to control values are denoted by asterisks: *p < 0.05. **(D)** Summary bar graphs showing the mean nociceptive scores in the dentin-exposed group of rats without any treatment (control; withe columns) and their minimum values (during the entire period of administration) in dentin-exposed rats treated with A-317491 (*n* = 6), mefloquine (*n* = 7) and ^10^PANX (*n* = 8) (black columns). Each bar represents mean ± SE of the number of experiments. Significant differences between columns (shown by solid lines) are denoted by asterisks: *p < 0.05.

**FIGURE 4:**
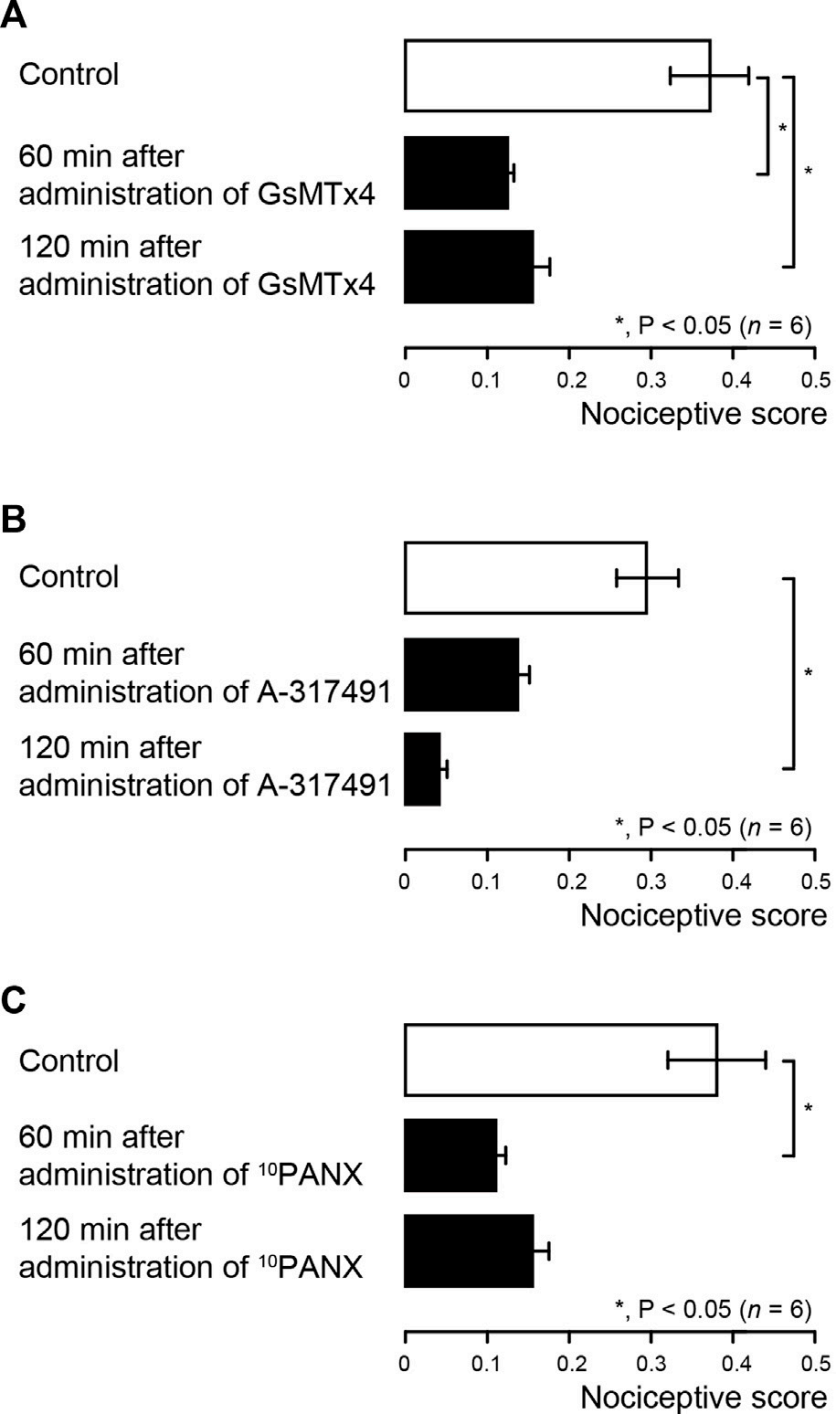
Pharmacological effects of Piezo1 channel (GsMTx-4), PANX-1 (^10^PANX) and P2X_3_ receptor (A-317491) antagonists on dentinal sensitivity in rat first molar **(A–C)** Bar graphs showing the mean nociceptive scores following cold water (0.1 ml, 4.0–7.0°C) stimulation to the dentin surface of the lower first molar in the dentin-exposed group rats without any treatment (control; withe columns) and their values treated with GsMTx-4 (A; *n* = 6), A-317491 (B; *n* = 6), and ^10^PANX (C; *n* = 6) (black columns) 60 min (middle columns) and 120 min (lower columns) after administration of each agent. The cold water stimuli-induced nociceptive scores were measured repeatedly at 60 and 120 min later each (see also Materials and Methods). Each bar represents the mean ± SE of the number of experiments. Asterisks denote significant differences between columns (shown by solid lines): *p < 0.05.

The lower incisors and molars were stimulated to induce dentinal sensitivity by applying cold water (0.1 ml, 4.0–7.0°C). The procedures for evaluating nociceptive scores by dentinal sensitivity were modified from the methods described by Chidiac et al. (2002), whereas behavioral scoring methods (see below and Figure 1D-1–D-4) were almost identical to the methods. To evaluate nociceptive scores caused by prolonged and long-lasting “tooth pulp inflammatory pain (as pulpal pain),” Chidiac et al. (2002) calculated the nociceptive scores at the end of the 60 min observation period by blocks, 3 min each, following one-shot intradental application of irritants (capsaicin or formalin) to the incisors. In the present study, however, we aimed to evaluate nociceptive scores caused by acute “dentinal sensitivity” as the first pain. For this purpose, we measured and calculated the nociceptive scores for the 180 s observation period following cold water (0.1 ml, 4.0–7.0°C) stimulation of the tooth surface (i.e., etched enamel surface, or etched dentin-exposed surface without any treatment of the surface or with a bonding agent applied to the dentin surface) of the rat incisors or molar, or mouse incisors. For a series of experiments to examine the time-dependent effects of pharmacological agents (see below) on evoked-dentinal sensitivity, we obtained nociceptive scores as control values before any systemic administration of the pharmacological agents to the dentin-exposed group of rats. We administered the pharmacological agent to the rat several minutes after measuring the control value of the nociceptive score by applying a cold water stimulus. We then repeatedly applied cold water to the exposed dentin surface at 10, 60, 120, 180, 240, 300, and 360 min later each for incisors and measured/calculated the nociceptive scores for the 180 s observation period following cold water stimulation. Thus, we obtained cold water stimuli-induced nociceptive scores at each 10, 60, 120, 180, 240, 300, and 360 min following the systemic application of pharmacological agents. For molars, same as the nociceptive score evaluation for incisors, the cold water stimuli-induced nociceptive scores were measured and calculated repeatedly at 60 and 120 min each following systemic application of pharmacological agents. We carefully observed rat/mouse behavior and measured nociceptive scales to evaluate the nociceptive scores of the evoked-dentinal sensitivity for 180 s after stimulation. Nociceptive behavior was classified on a scale of 0–3, and the nociceptive score was calculated based on the method described by Chidiac et al. (2002) (Figure 1D-1–D-4).

### Experimental conditions

For the behavioral analysis, cold stimuli (0.1 ml, 4.0–7.0°C when applied on the dentin or enamel surface) were applied to the etched enamel surfaces without dentin exposure in the rats and mice of the “control group.” In the “dentin-exposed group,” rats and mice were subjected to cold stimuli on the exposed dentin surface and further divided into the following eight groups: 1) rats and mice without treatment of the dentin surface and rats treated with 2) a bonding agent (Sun Medical Company, Ltd.) on the dentin surface, 3) a P2X_3_ receptor-selective antagonist [A-31749; 0.3 nmol/g (weight), intravenously (i.v.)], 4) a non-selective PANX-1 channel blocker [mefloquine; 0.03 mg/g (weight), orally], 5) a selective PANX-1 channel blocker [^10^PANX; 2.58 nmol/g (weight), i.v.], 6) a selective Piezo1 channel blocker [GsMTx-4; 0.8 nmol/g (weight), i.v.], 7) a TRPC5 channel antagonist [HC-070; 0.008 nmol/g (weight), i.v.], and 8) a TRPA1 channel antagonist [HC-030031; 8 nmol/g (weight), i.v.]. The dose for systemic administration was selected such that the final concentration was 3.8 μM for A-31749, 32 μM for ^10^PANX, 10 μM for GsMTx-4, 100 nM for HC-070, and 100 μM for HC-030031 in the estimated total blood volume. The series of concentrations used in this study was determined according to a previous study (Sato et al., 2015, 2018; Shibukawa et al., 2015; Kimura et al., 2016; Bernal et al., 2021). DTR- and Col1 (2.3)-Cre/DTR-mice, as well as a rat, were randomly selected for each experiment. Cages of animals for experimental group assignment were randomized to reduce environmental stress influencing nociceptive processing. Each experiment was to initiate a habituation period and complete a behavioral test from 14:00 to 21:00 each day. The order of the drug/bonding treatments and measurements was also randomized. The administration of drugs or application of bonding agents were performed using a single-blinded method.

### Reagents

The stock solutions of the reagents used included A-317491, a P2X_3_ receptor antagonist (Sigma-Aldrich); ^10^PANX, a selective PANX-1 blocker; and GsMTx-4, a selective Piezo1 channel blocker (R&D Systems, Inc, Minneapolis, United States) were prepared in sterilized PBS (Life Technologies, Ltd, Paisley, United Kingdom) and diluted with physiological saline (Otsuka Pharmaceutical Co., Ltd., Tokyo, Japan) to the appropriate concentration for administration. Stock solutions of the TRPA1 channel antagonist, HC-030031 (Abcam), and TRPC5 channel antagonist, HC-070 (MedChemExpress, Monmouth Junction, NJ, United States), were prepared in DMSO, and diluted with physiological saline to the appropriate concentration for administration. Mefloquine, a non-selective PANX-1 blocker, was purchased from Hisamitsu Pharmaceutical Co., Inc, and Tokyo Chemical Industry Co., Ltd (Tokyo, Japan). Because this reagent is water-insoluble, it was administered orally with physiological saline. Anesthesia was induced with 3% isoflurane (FUJIFILM Wako Pure Chemical Co., Osaka, Japan).

### Electrophysiological recordings of trigeminal nerve activities by dentinal sensitivity

Wistar rats were anesthetized with 3% isoflurane (FUJIFILM Wako Pure Chemical Co.) and pentobarbital sodium (Nacalai Tesque, 400 mg/kg, i.p.). Decapitation was performed under anesthesia. Skin from the top of the head and connective tissue was removed to expose the skull, followed by decerebration surgery to access the TG directly. The right trigeminal nerve was made directly visible under a stereoscopic microscope, free from surrounding tissues after the decerebrate, and cut at the point of its central side. A dissected and isolated whole TG nerve fibers were lifted onto an Ag-AgCl electrode, and an indifferent electrode was placed in nearby tissue. For the dentin-exposed group rats, the dentin cold stimulation-induced neural activities were amplified (K-1; Iyodenshikagaku, Nagoya, Japan) and recorded on a computer using a PowerLab system (PowerLab/sp4; AD Instruments, Bella Vista, NSW, Australia). For stimulation, the right first molar following dentinal exposure preparation (see above) was covered by a tip of a 2.5 ml syringe fixed with a flexible arm. To adapt to body temperature, 37°C distilled water was applied by the syringe before stimulation. After 30 s of 37°C stimulation, cold distilled water (4.0–7.0°C) was applied at 1.5 ml, and this amount induced a quick change of temperature with minimum tactile sensation from the water. After 30 s after the cold stimulus onset, 37°C distilled water was applied again. For data analysis, we calculated the net average frequency for 10 s after the stimulus onset, which was obtained by subtracting the spontaneous frequency for the 10 s duration before stimulation from after stimulation, according to the previously described methods (Yasumatsu et al., 2003, 2020). To evaluate pharmacological effects on the cold stimulation-induced neuronal activities of the TG nerve, we administrated the following agents to the rats 74 min (73.5 ± 15.1 min) before the recordings: 1) a P2X_3_ receptor-selective antagonist [A-31749; 0.3 nmol/g (weight), i.v.], 2) a selective PANX-1 channel blocker [^10^PANX; 2.58 nmol/g (weight), i.v.], 3) a selective Piezo1 channel blocker (GsMTx-4; 0.8 nmol/g (weight), i.v.], or 4) saline [1.3 μL/g (weight), i.v.].

### Statistical analyses

Data are expressed as mean ± standard error (S.E.), and *n* represents the number of rats/mice tested. Nociceptive scores and neural activities were evaluated using the Kruskal-Wallis test [non-parametric analysis of variance (ANOVA)], followed by Dunn’s multiple comparison test. The use of non-parametric ANOVA is justified by the fact that reported nociceptive scores are ordinal entities or categories (Chidiac et al., 2002). The non-parametric Mann-Whitney *U* test was also applied to analyze the nociceptive score between the groups of DTR- and Col1 (2.3)-Cre/DTR-mice. Statistical significance was set at *p* < 0.05. All statistical analyses were performed using GraphPad Prism version 7 (GraphPad, Inc, La Jolla, CA, United States). Sample size estimation was performed using the data obtained from our pilot study. The difference in nociceptive score between the control group and the dentin-exposed group was 0.17, and the SD was set at 0.08. Five samples were required to detect a difference in nociceptive behavior with a type I error of 0.05, and a power of 0.8 in a two-tailed unpaired *t*-test. A total of 5–10 animals were included to account for the possibility of withdrawal from the study.

## Results

### Nociceptive scores from a rat model with and without dentin exposure

The lower incisors of rats in the control and dentin-exposed groups were stimulated with cold water (0.1 ml, 4.0–7.0°C when applied to the tooth surface). In control group rats, the nociceptive score was 0.03 ± 0.01 (*n* = 8). The score of rats in the dentin-exposed group treated with a bonding agent was 0.07 ± 0.02 (*n* = 8); there were no significant differences in scores between the control group and the group subjected to treatment with a bonding agent (Figure 2A). However, rats in the dentin-exposed group without any treatment (but with an etched dentin surface) had significantly higher scores (0.23 ± 0.04, *n* = 10) than those of rats in both the control and bonding agent-treated dentin-exposed groups (Figure 2A).

### Time-dependent effects of pharmacological agents on evoked-dentinal sensitivity

To evaluate whether mechanosensitive Piezo1 channels and PANX-1 in odontoblasts and P2X_3_ receptors in TG neurons are necessary to evoke dentinal sensitivity, we evaluated the time-dependent effects of antagonists of these receptors/channels on the nociceptive scores induced by dentin cold stimuli in rats in the dentin-exposed group. For a series of experiments, prior to any systemic administration of the pharmacological agents to the dentin-exposed group of rats, we obtained nociceptive scores as control values (white circles in Figures 2B–D, 3A–C). We administered the pharmacological agent to the rat several minutes after measuring the control value of the nociceptive score by applying a cold-water stimulus. We then repeatedly measured the cold water stimuli-induced nociceptive scores at 10, 60, 120, 180, 240, 300, and 360 min later each (black circles in Figures 2B–D, 3A–C; see also Materials and Methods).

Systemic administration of a Piezo1 channel blocker (GsMTx-4; Figure 2B), P2X_3_ receptor antagonist (A-317491; Figure 3A), a non-selective PANX-1 blocker (mefloquine; Figure 3B), and selective PANX-1 blocker (^10^PANX; Figure 3C) significantly and time-dependently decreased nociceptive scores in groups with dentinal sensitivity. Administration of GsMTx-4 significantly decreased the nociceptive score to 0.05 ± 0.01 (*n* = 7; Figure 2E) and 0.06 ± 0.01 (*n* = 7) at 10 and 60 min after administration, respectively, compared to the control value of 0.23 ± 0.03 (*n* = 7) (Figures 2B,E). When A-317491 was administered to dentin-exposed rats, the nociceptive score significantly declined to 0.05 ± 0.02 (*n* = 6) at 120 min after administration, compared to the control value of 0.32 ± 0.09 (*n* = 6; Figures 3A,D). When mefloquine was administered to rats in the dentin-exposed group, the nociceptive score was significantly reduced to 0.06 ± 0.02 (*n* = 7) and 0.06 ± 0.01 (*n* = 7; Figure 3B) at 60 and 120 min after administration, respectively, compared to rats not treated with mefloquine (0.26 ± 0.03, *n* = 7) (Figures 3B,D). In addition, for dentin-exposed rats, injection of ^10^PANX led to a significantly decreased score of 0.07 ± 0.01 (*n* = 8) at 60 min after administration, compared to the control value of 0.24 ± 0.04 (*n* = 8; Figures 3C,D).

To evaluate whether temperature-sensitive TRPC5 channels (Bernal et al., 2021), and temperature-/pH-/mechano-sensitive TRPA1 channels (Tsumura et al., 2012; Kimura et al., 2016) on odontoblasts contribute to the sensory transduction sequence in dentinal sensitivity, we also evaluated the time-dependent effects of TRPC5 and TRPA1 channel antagonists on nociceptive scores following cold stimuli application in rats in the dentin-exposed group. Systemic administration of the TRPC5 channel blocker (HC-070) did not lead to any significant reduction in the nociceptive scores of groups with dentinal sensitivity during the entire period of its application (Figure 2C); the scores were 0.14 ± 0.02 (*n* = 8; Figure 2E) at 60 min after administration, compared with scores without administration (0.23 ± 0.03, *n* = 8) (Figures 2C,E). TRPA1 receptor antagonist administration (HC-030031; Figure 2D), however, slightly, but significantly, decreased the nociceptive score to 0.15 ± 0.01 (*n* = 8; Figure 2E) at 60 min after administration, compared with the score without administration (0.26 ± 0.03, *n* = 8) (Figures 2D,E).

### Effects of Piezo1 channels, PANX-1, and P2X_3_ receptor antagonists on evoked-dentinal sensitivity in rat first molar

We also evaluated the contribution of Piezo1 channel, PANX-1 and P2X_3_ receptor activations to the generation of cold water (0.1 ml, 4.0–7.0°C)-induced dentinal sensitivity in lower first molars of dentin-exposed rats, *via* intercellular signaling between odontoblast and TG neuron. The cold-water stimuli-induced nociceptive scores were measured repeatedly at 60 and 120 min later each (see also Materials and Methods). Systemic administration of Piezo1 channel blocker (GsMTx-4) significantly decreased the nociceptive score to 0.12 ± 0.01 and 0.15 ± 0.02 at 60 and 120 min after administration, respectively, compared to the control value of 0.37 ± 0.05 (Figure 4A; *n* = 6). The nociceptive score significantly declined to 0.04 ± 0.01 at 120 min after administration of P2X_3_ receptor antagonist (A-317491) to dentin-exposed rats, compared to the control value of 0.30 ± 0.04 (Figure 4B; *n* = 6). Injection of ^10^PANX into the dentin-exposed rats also showed a significant decrease in the score of 0.11 ± 0.01 at 60 min after administration, compared to the control value of 0.38 ± 0.06 (Figure 4C; *n* = 6).

### Colocalization of A-neuron marker protein and P2X_3_ receptors at the dentin-pulp border of rat lower incisors and molars

**FIGURE 5:**
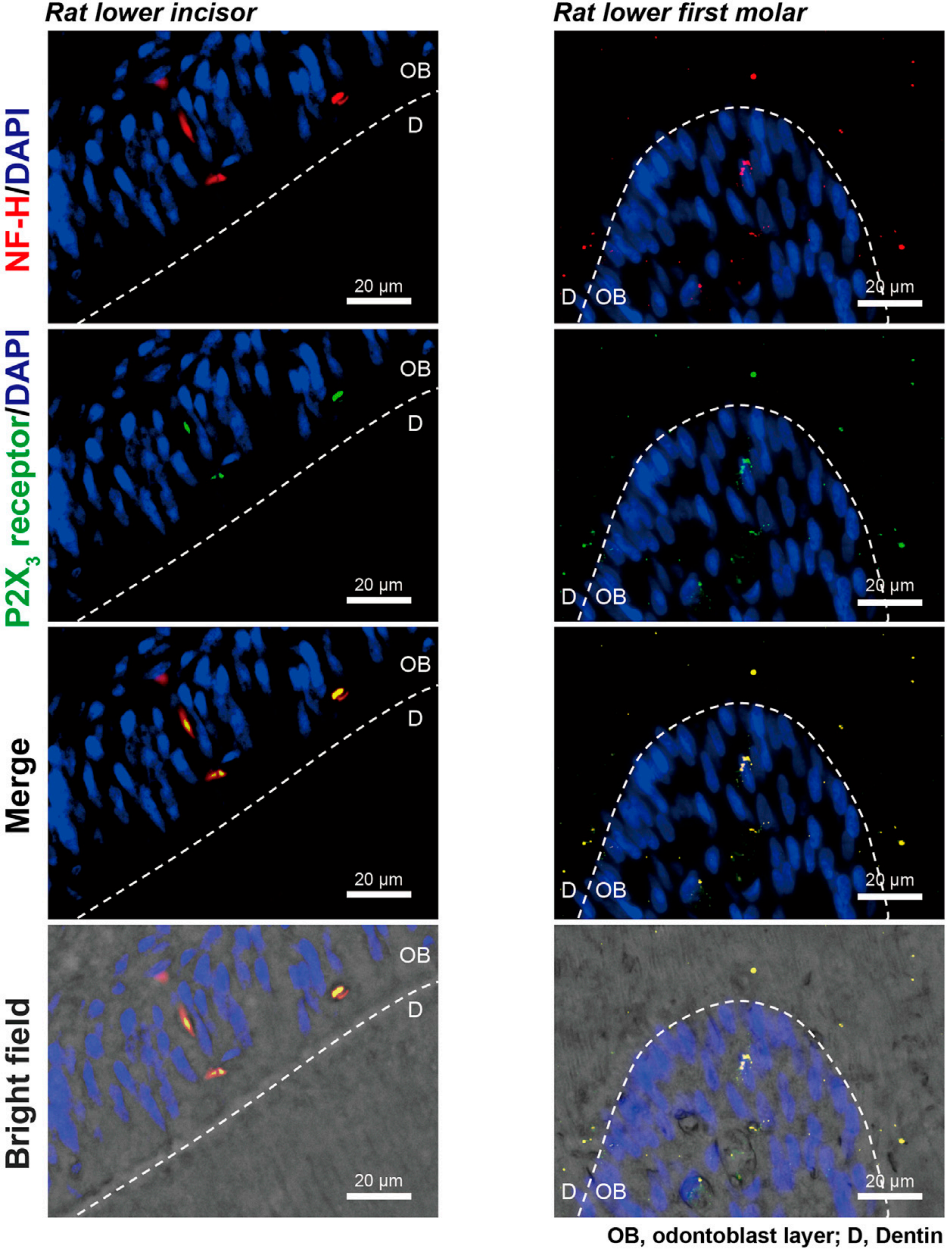
Expression of A-neuron marker protein and P2X_3_ receptors at the dentin-pulp border of rat lower incisors and molars. Immunofluorescence analysis showed immunoreactivities for myelinated A-neuron marker protein, NF-H (red in upper, second lower, and lower panels), and ionotropic ATP receptor subtype, P2X_3_ receptor (green in second upper, second lower, and lower panels), in the dentin-pulp border of rat lower incisors (left panels) and molars (right panels). Nuclei are shown in blue (DAPI). Scale bars, 20 μm.

In the immunofluorescence analysis, we observed colocalization of immunoreactivities for myelinated A-neuron marker protein, NF-H, and ionotropic ATP receptor subtype, P2X_3_ receptor, in the odontoblastic and sub-odontoblastic regions of rat lower incisors (left panels) and molars (right panels; Figure 5). In the incisors, NF-H and P2X_3_ receptors were colocalized at the odontoblast layer, while they were expressed not only at the odontoblast layer but also inside dentinal tubules in the molars.

### Dentinal sensitivity was eliminated in mice that were allowed odontoblast-specific depletion by somatic Cre-mediated gene recombination

**FIGURE 6:**
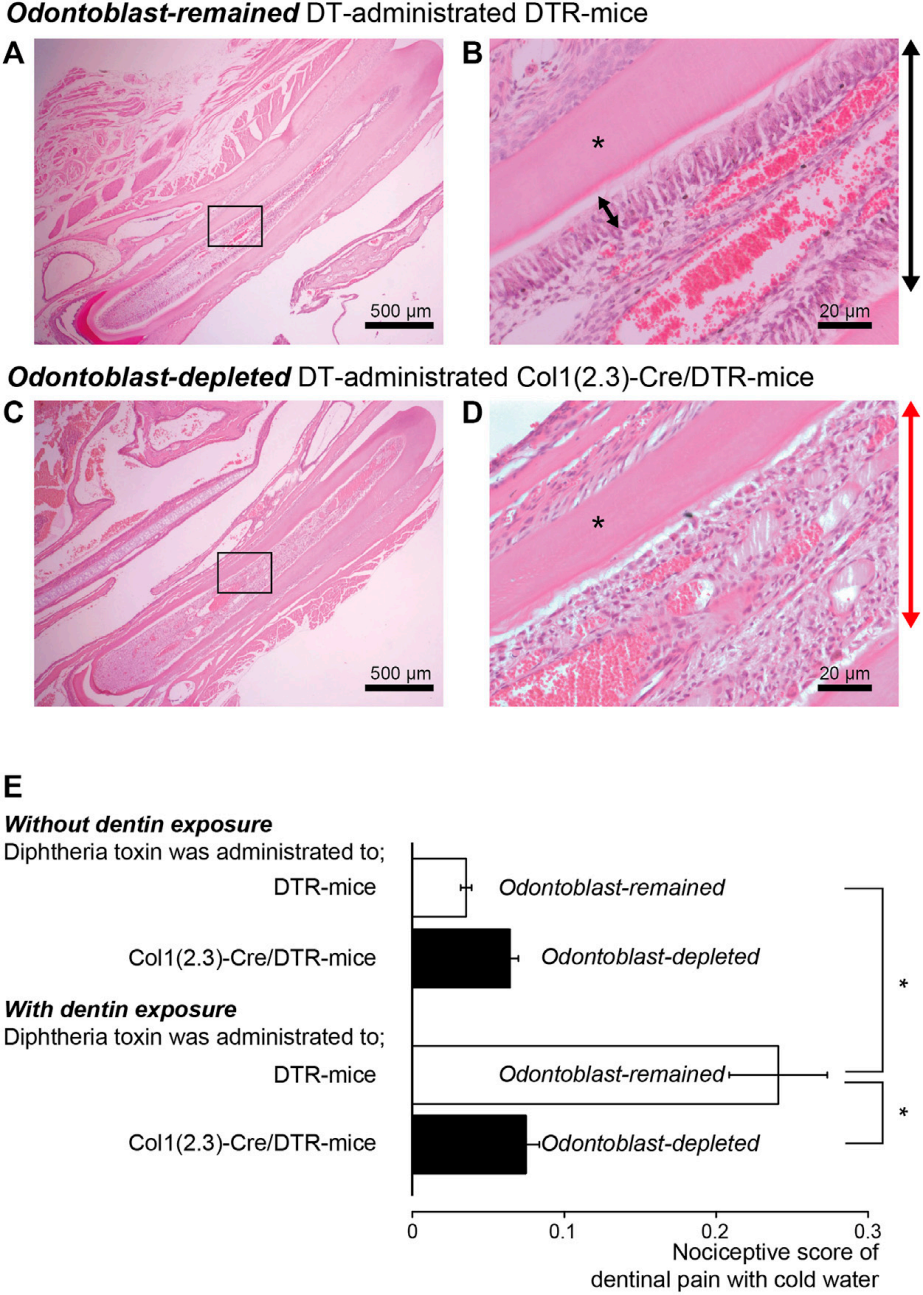
Reduction in nociceptive scores in dentin-exposed mice with odontoblast-specific depletion by somatic Cre-mediated gene recombination **(A,B)** Morphological observations revealed highly polarized odontoblast arranged at the dentin-pulp border in DTR-mice. **(C,D)** In Col1 (2.3)-Cre/DTR-mice exhibiting somatic odontoblast-specific depletion upon administration of diphtheria toxin (DT), we could not observe highly polarized odontoblast arranged at the dentin-pulp border. Bars indicate 500 μm in **(A,C)**, and 20 μm in **(B,D)**. Images in **(A–D)** were obtained from both mice after 1 week of 250 ng DT intraperitoneal injection every 24 h. Photos of **(B,D)** show the areas shown by squares in A and C. **(E)** Bar graph shows nociceptive scores following cold water stimulation without (upper two columns) or with (lower two column) dentin-exposure in control DTR-mice (open columns; *n* = 8) and odontoblast-deficient Col1 (2.3)-Cre/DTR-mice (filled column; *n* = 8). Each bar shows the mean ± S.E. Significant differences between columns (shown by solid lines) are denoted by asterisks: *p < 0.05.

To evaluate the specific role of odontoblasts in generating dentinal pain as sensory receptor cells, we administered DT every 24 h for 1 week to DTR- and Col1 (2.3)-Cre/DTR-mice. Morphological observations of the hematoxylin-eosin-stained dental pulp from Col1 (2.3)-Cre/DTR- and DTR-mice before measuring nociceptive scores revealed highly polarized odontoblasts arranged at the dentin-pulp border (Figures 6A,B) in DTR-mice (as control), but not in Col1 (2.3)-Cre/DTR-mice, allowing somatic odontoblast-specific depletion by DT administration (Figures 6C,D).

In mice in the control group, in which the enamel surface was etched (dentin was not exposed), the nociceptive scores were 0.04 ± 0.004 (*n* = 8) in DTR-mice and 0.07 ± 0.01 (*n* = 8) in Col1 (2.3)-Cre/DTR-mice, showing no significant difference (upper two columns in Figure 6E). The nociceptive scores in the dentin-exposed group were 0.24 ± 0.03 (*n* = 8) in DTR-mice and 0.08 ± 0.01 (*n* = 8) in odontoblast-deficient Col1 (2.3)-Cre/DTR-mice, showing significant differences in scores between both groups (lower two columns in Figure 6E). The nociceptive scores in odontoblast-depleted Col1 (2.3)-Cre/DTR-mice did not differ significantly between the control and dentin-exposed groups. In addition, we could observe nothing significant differences in values of the nociceptive score between the dentin-exposed group of odontoblast-deficient Col1 (2.3)-Cre/DTR-mice and the control group of odontoblast-remained DTR-mice, during application of cold water stimuli. These score values in odontoblast-depleted Col1 (2.3)-Cre/DTR-mice of the dentin-exposed group might be an effect of somatic sensation by water application as baseline values.

**FIGURE 7:**
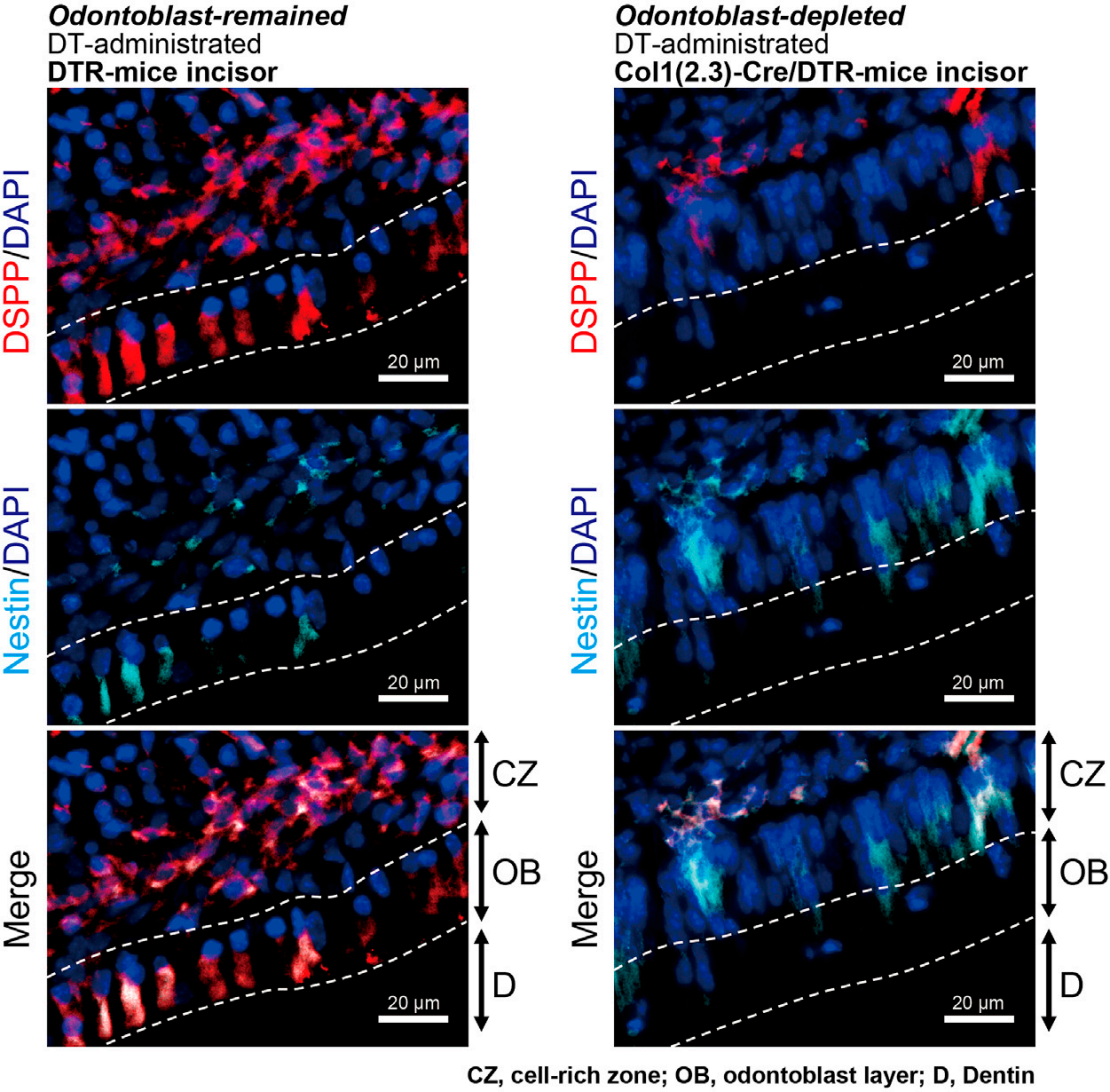
Observation of odontoblast-specific makers in incisors of DT-injected DTR-mice and Col1 (2.3)-Cre/DTR-mice after performing a series of measurements of nociceptive scores. Immunofluorescence analysis using DT-administered DTR-mice (as control; left panels) showed that DSPP-positive cells (red) were localized in both the cell-rich zone (CZ) and odontoblast (OB) layer in proximity to dentin (D). Nestin-positive odontoblasts (blue) were also observed in both CZ and OB (left panels). Analysis using Col1 (2.3)-Cre/DTR-mice (right panels) showed that DSPP-positive (red) and nestin-positive cells (blue) were not observed in the OB layer. Scale bars, 20 μm.

After measuring the nociceptive scores, we analyzed dentin sialophosphoprotein (DSPP; red in Figure 7) and nestin (blue in Figure 7) immunoreactivities in cells in the dentin-pulp border of the dental pulp of both odontoblast-deficient Col1 (2.3)-Cre/DTR-mice and DTR-mice. Highly polarized DSPP-/nestin-positive odontoblasts were observed in the DTR-mice with intact odontoblasts (left panels in Figure 7) even after cavity preparation in the incisor and measurement of nociceptive scores. We could not observe cells showing DSPP-/nestin-immunoreactivities just beneath the dentin in the odontoblast-deficient Col1 (2.3)-Cre/DTR-mice (right panels in Figure 7), in addition to their nuclei (dark blue in Figure 7).

### Colocalization of A-neuron marker protein and P2X_3_ receptors at dentin-pulp border of DT-administrated DTR- and col1 (2.3)-Cre/DTR-mice

**FIGURE 8:**
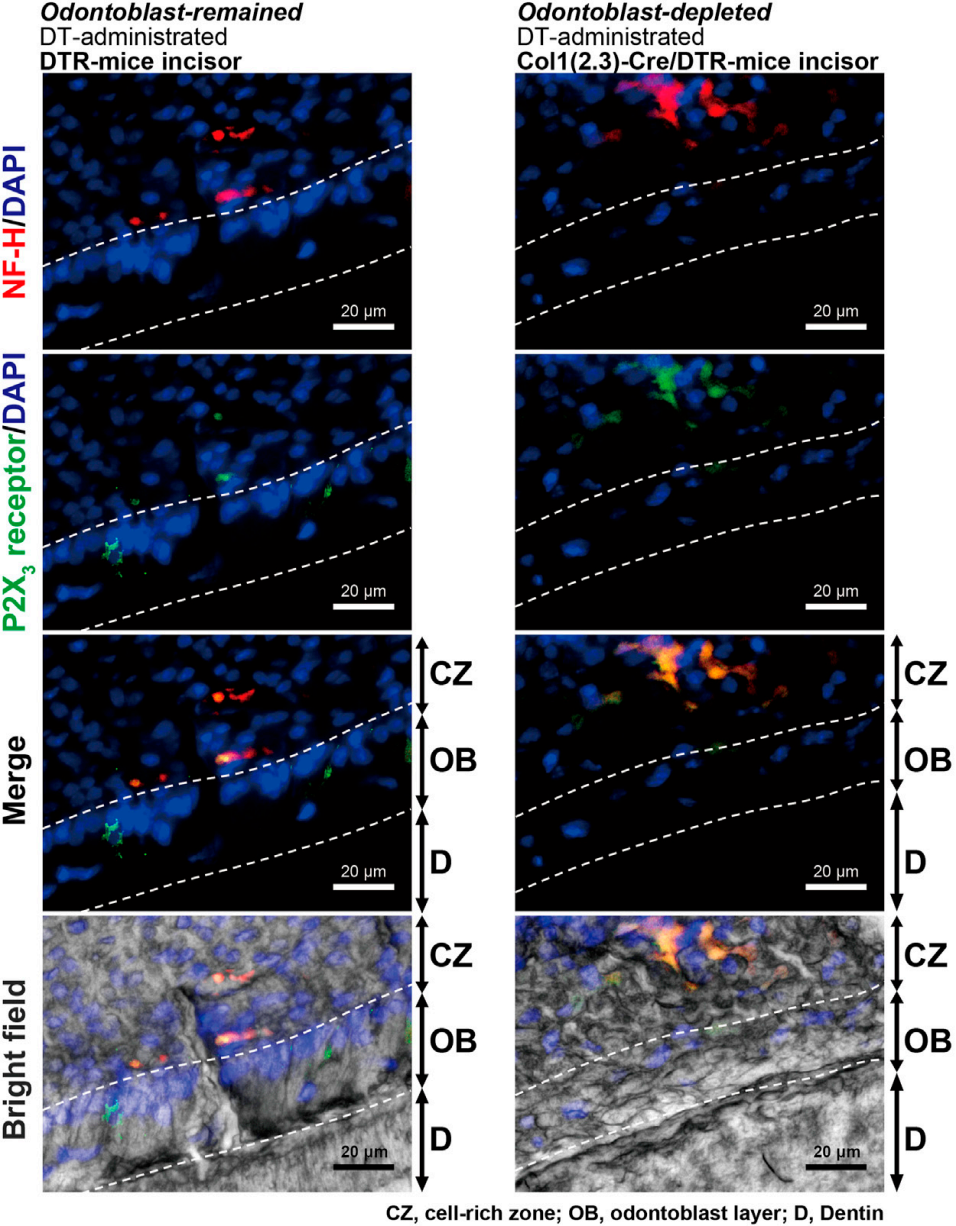
Observation of A-neuron marker protein and P2X_3_ receptors in incisors of DT-injected DTR-mice and Col1 (2.3)-Cre/DTR-mice after performing a series of measurements of nociceptive scores. Immunofluorescence analysis showed that both DT-administered DTR-mice (left panels) and Col1 (2.3)-Cre/DTR-mice (right panels) expressed myelinated A-neuron marker protein, NF-H (red in upper, second lower, and lower), and ionotropic ATP receptor subtype, P2X_3_ receptor (green in second upper, second lower and lower), in the sub-odontoblastic region of the lower incisors. CZ, cell-rich zone; OB, odontoblast layer; and D, dentin. Nuclei (DAPI) are shown in blue. Scale bars, 20 μm.

In the immunofluorescence analysis, we also observed colocalization of immunoreactivities for myelinated A-neuron marker protein, NF-H, and ionotropic ATP receptor subtype, P2X_3_ receptor, in the sub-odontoblastic region of the lower incisors of DT-administrated DTR- (left panels) and Col1 (2.3)-Cre/DTR-mice (right panels; Figure 8), indicating that P2X_3_ receptor-positive A-neurons were morphologically intact in both the odontoblast-remained DTR- and odontoblast-eliminated Col1 (2.3)-Cre/DTR-mice.

### Action potential recordings from the whole TG during cold water stimulation to the exposed dentin surface of the lower first molar

**FIGURE 9:**
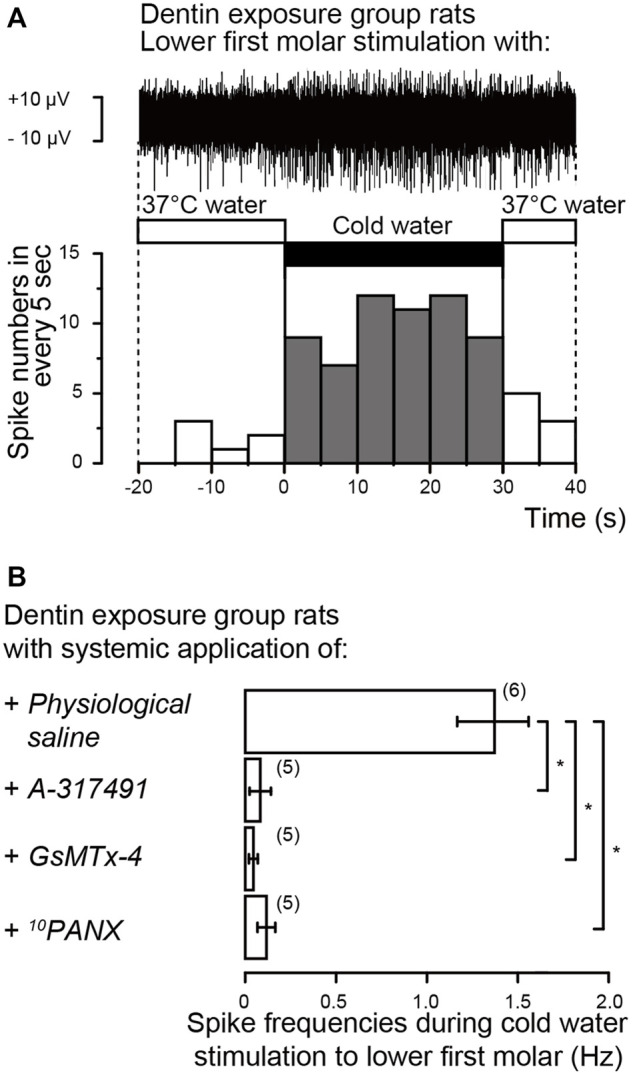
Electrophysiological recordings from trigeminal ganglion (TG) neurons **(A)** Representative trace (upper) and its spike histogram showing spike number in every 5 s (lower) of event-related neuronal activities from the whole TG neurons following cold water (middle black box) or warm water (middle open boxes) stimulation to exposed dentin surface of lower first molars in rat injected by physiological saline intraperitoneally. The stimulated dentin was adapted to warm (37.0°C) distilled water, and the cold (4.0–7.0°C) stimulus was applied for ∼30 s **(B)** Averaged spike frequencies in response to cold water stimuli to exposed dentin surface of lower first molars in the rat following intraperitoneal injection of physiological saline (upper; *n* = 6), A-317491 (second upper; *n* = 5), GsMTx-4 (second lower; *n* = 5) or ^10^PANX (lower; *n* = 5). Each bar represents the mean ± SD of the number of experiments. Asterisks denote significant differences between columns (shown by solid lines): *p < 0.05.

We further recorded event-related neuronal activities from the whole TG neurons following cold water (4.0–7.0°C) or warm water (37.0°C) stimulation (1.5 ml) to the lower first molars. In the rat injected by physiological saline intraperitoneally, we could observe an increase in the spike frequencies recorded form whole TG during cold water application to the exposed dentin surface of the lower first molars, compared to those by warm water stimulation (upper trace in Figure 9). In the same condition, we could not observe any spike frequency increases from whole TG during cold water application compared to those during warm water stimulation, when the dentin of the lower first molars was not exposed (not shown). During the recording of event-related whole TG neuron activities, we could observe the following three spike units; 1) the cold stimulus-responded neurons showing spike duration (width) of ∼2 ms without any adaptation, 2) the neurons showing ∼3 ms spike duration responding to warm water, and 3) the neurons showing quick adaptation which may respond tactile stimuli. Among them, we further analyzed cold stimulus-responded neurons. The cold stimulus- (i.e., dentinal sensitivity-) responded neurons showed high spike frequency specifically and continuously responded to cold stimulus for 30 s after stimulus onset (lower graph, Figure 9A). As shown in Figure 9B, the impulse frequencies of the neuron during cold water application to the exposed dentin surface were almost completely and significantly abolished by intraperitoneal injection of A-317491, GsMTx-4, or ^10^PANX.

## Discussion

In the present study, we evaluated the nociceptive score in dentinal sensitivity by stimulating the lower incisors of rats and mice, and the molars of rats with cold water. Systemic administration of antagonists of Piezo1 and TRPA1 channels and PANX-1 and P2X_3_ receptors significantly and time-dependently reduced nociceptive scores induced by dentinal sensitivity in rats of the dentin-exposure group compared to those without systemic antagonist administration. These results indicate the involvement of Piezo1/TRPA1–PANX1–P2X_3_ axis activation in the development of dentinal sensitivity. Cold stimuli applied to the exposed dentin surface of lower molars also enhanced spike frequencies in the TG neurons, while the administration of antagonists for the Piezo1–PANX1–P2X_3_ axis reduced the frequencies. However, it is well known that these receptors and channels are ubiquitously expressed in various tissues in a wide variety of cells (Bagriantsev et al., 2014; Khakh et al., 2001) and related to the sensory system, not only peripheral but also the central nervous system. Thus, we further examined whether odontoblasts are necessary for dentinal sensitivity. The nociceptive score in the dentin-exposed group with odontoblast deficiency (Col1 (2.3)-Cre/DTR-mice) was significantly lower than that in DTR-mice. Together, these results show that odontoblasts are sensory receptor cells capable of detecting cellular deformation, due to hydrodynamic force inside dentinal tubules, *via* mechanosensitive Piezo1/TRPA1 channels and that ATP released from odontoblasts by PANX-1 acts as the predominant neurotransmitter for P2X_3_ receptors on pulpal neurons to drive the sensory transduction sequence for dentin.

In addition, we observed no significant difference in the scores between rats in the dentin-exposed group subjected to bonding agent treatment on the dentin surface and the control group, in which the enamel surface was etched but dentin was not exposed, indicating that dentinal fluid movement is necessary to induce dentinal pain by cold stimuli (Andrew and Matthews, 2000; Närhi M, 2003; Charoenlarp et al., 2007; Aminoshariae and Kulild, 2021; Held et al., 2021). The nociceptive painful sensation of the dentin-pulp complex is conducted by two different sets of dental pulp afferents in TG neurons: 1) myelinated Aδ neurons innervating the dental pulp periphery and 2) unmyelinated C fibers located in the deeper parts of the dental pulp (Närhi M, 2003; Aminoshariae and Kulild, 2021). The Aδ fibers mediate sharp dental pain (as the first pain) responsible for “dentinal pain” (see also Introduction), while C neurons mediate dull pain (as the second pain) responsible for “pulpal pain” occurring in teeth with inflamed dental pulp (i.e., pulpitis); low-intensity/frequency electrical stimulation to the tooth surface also induces non-painful sensation (termed “prepain”) caused by activation of Aβ dental pulp afferents of TG neurons (Närhi M, 2003; Kubo et al., 2008; Aminoshariae and Kulild, 2021). A recent study implied that TRPC5 channels in odontoblasts may contribute to the generation of “inflammatory tooth pain” in teeth with pulpitis during direct cold stimulation of the dental pulp but not dentin surface (Bernal et al., 2021). In our present study, however, TRPC5 channel antagonist administration had no significant effects on nociceptive scores following cold stimulation of the exposed dentin surface in the dentin-exposed group rats; in contrast, Piezo1 channel antagonist administration almost completely abolished nociceptive behavior following stimulation of the exposed dentin surface. These results indicate that the contribution of TRPC5 channels in odontoblasts to the generation of dentinal sensitivity by various stimuli to the exposed dentin surface is unlikely. However, we could not exclude the possible contribution of TRPC5 channels on the generation of pain from direct cold stimulation of the “inflamed” tooth pulp, but not dentinal sensitivity including “dentin hypersensitivity” (Held et al., 2021).

It has been reported that lacked nerve fibers passed through the odontoblast layer and innervated into the dentin (Nishikawa, 2007) in the rodent incisors, while recent convergent results have indicated that the nerves are essential for dental growth (Kaukua et al., 2014; Krivanek et al., 2017). In both the incisors and molars of rats, we could observe the expression of A-neuron marker protein (NF-H) and P2X_3_ receptors double-positive neurons in the odontoblastic and sub-odontoblastic regions. In addition, we showed the immunoreactivity of NF-H and P2X_3_ receptors in both the odontoblast-remained DTR- and odontoblast-eliminated Col1 (2.3)-Cre/DTR-mice incisors, indicating that P2X_3_ receptor-positive A-neurons were morphologically intact in both, only odontoblasts were somatically depleted in the Col1 (2.3)-Cre/DTR-mice. The results indicated again that odontoblasts are necessary for generating dentinal pain. In addition, we have shown that generation of an action potential in the Aδ afferents of TG neurons by activation of P2X_3_ receptors upon ATP release from mechanically stimulated odontoblasts (Sato et al., 2018). Therefore, intercellular signal communication from odontoblast to NF-H- and P2X_3_ receptor-positive A-neurons in both the incisor and molar established by the Piezo1/TRPA1 channel-PANX-1-P2X_3_ receptor complex plays a significant role in the generation of dentinal pain.

In the odontoblast-eliminated Col1 (2.3)-Cre/DTR-mice, we could observe a marked reduction of DSPP-positive cells (see Figure 7), while increases in the nestin-positive cells in the cell-rich zone (CZ) of incisor, compared to the odontoblast-remained DTR-mice. It has been reported that after the elimination of odontoblast in Col1 (2.3)-Cre/DTR-mice by DT, CZ-localizing nestin-positive, and nestin-negative cells proliferate and differentiate into odontoblast-like cells in response to odontoblast depletion (Zhao et al., 2021). In the odontoblast-eliminated Col1 (2.3)-Cre/DTR-mice incisors, we could also observe increases in the expression of not only nestin but also other pluripotent stem cell protein(s) (personal communications by TO and YS). Although further study will be needed, pluripotent stem cell protein-positive cells in CZ might be capable of immediately proliferating and differentiating into odontoblasts after dentin injury as a locally differentiated mechanism to regenerate dentin (regenerative dentin formation).

The mechanisms underlying dentinal sensitivity have been classically described by three hypotheses: 1) “neural (free nerve ending) theory,” 2) “hydrodynamic theory (see Introduction),” and 3) “odontoblast transducer (or receptor) theory” (Bränström, 1963; Ten Cate, 1994). The “neural theory” hypothesizes that free nerve endings entering the dentinal tubules directly detect stimuli applied to the dentin, while the “hydrodynamic theory” assumes that the nerve terminals indirectly detect them as mechanical stimuli due to dentinal fluid movement (hydrodynamic force) inside dentinal tubules. Recent findings suggest that medium-to large-sized primary afferents containing neuropeptides innervating the dental pulp (dental primary afferent neurons) possess Piezo2 channels (Won et al., 2017). The nerve endings do not penetrate the dentinal tubules; however, they extend to the inner third of the tubules (Sessle, 1987). Thus, the nerve endings do not reach the EDJ. Despite the lack of nerve endings, the EDJ area is sensitive to external stimuli when the enamel is removed and the dentin is exposed. Merkel cells (MCs), which are touch receptors, form a part of the MC-neurite complex with sensory neurons through synaptic contact to mediate mechanosensory transduction. MCs release glutamate in response to cellular deformation through direct mechanical stimulation. The released glutamate activates N-methyl-D-aspartate receptors on sensory afferents (Higashikawa et al., 2019). Recent evidence has also shown that MCs and innervating Aβ sensory afferents, which both express Piezo2 channels, are necessary to mediate mechanically activated, slowly adapting responses. This has been referred to as a “two-receptor-site model,” which provides high spatial resolution for touch sensations such as those evoked by cutaneous mechanoreceptors (Woo et al., 2014). Piezo2 channels in dental primary afferent neurons, considered low-threshold mechanoreceptors (Fried et al., 2011), might also be involved in mechanosensory transduction by sensing dentinal fluid movement to develop dentinal sensitivity, similar to the two-receptor-site model. Based on our results, however, it is unlikely that both the “neural theory” and “hydrodynamic theory” explain the generation mechanism of dentinal sensitivity, as there were no differences in the nociceptive scores of DTR-mice without dentin exposure and Col1 (2.3)-Cre/DTR odontoblast-deficient mice with or without dentin exposure. These results showed that odontoblasts, not the nerve endings inside the dentinal tubules (excluding those that establish neural communication with odontoblasts), are necessary for the development of dentinal sensitivity.

The “odontoblast receptor theory” (Ten Cate, 1994) suggests that odontoblasts “directly” receive stimuli applied to the dentin surface *via* cellular processes inside the dentinal tubules. Convergent morphological studies have shown that odontoblast processes are limited to the inner one-to two-third of dentin (Lavicky et al., 2022), while Mahdee et al. (2018) described that odontoblast processes extend to the dentin-enamel junction. Although the distribution pattern of odontoblast processes in the dentinal tubules is controversial, the area without nerve endings and odontoblast processes inside tubules, neither of which reach the EDJ (Khatibi Shahidi et al., 2015), initiates painful responses following dentin stimulation. In addition, the area around the EDJ contains relatively few dentinal tubules and is often sensitive to stimulation. Together with the morphological/anatomical limitation, the “odontoblast transducer (receptor) theory” alone may not explain the generation of dentinal sensitivity (Bradley, 1995), and dentinal fluid movement inside dentinal tubules is necessary to induce dentinal pain (Andrew and Matthews 2000; Charoenlarp et al., 2007; Vongsavan and Matthews 2007; Lin et al., 2011). Based on the present results, odontoblasts act as mechanosensory receptor cells that may detect dentinal fluid movement through mechanical stimulation. Thus, the new model to describe development of dentinal sensitivity described by us can be thought of as an amalgamated model of the “odontoblast transducer (receptor) theory” and “hydrodynamic theory.” We could not exclude the possibility that odontoblasts may respond to direct mechanical stimulation applied to the dentin surface, in addition to dentinal fluid movement.

Recent evidence has shown that odontoblasts express various plasma membrane mechanosensitive ion channels, including TRPV1, TRPV2, TRPV4 (Son et al., 2009; Tsumura et al., 2012; Sato et al., 2013), TRPA1 (Tsumura et al., 2013), TRP-melastatin subfamily member-7 (Won et al., 2018), stretch-induced Ca^2+^ channels (Shibukawa and Suzuki, 1997), mechanosensitive K^+^ channels (Allard et al., 2000; Magloire et al., 2003), and Piezo1 and Piezo2 channels (Khatibi Shahidi et al., 2015; Sato et al., 2018; Miyazaki et al., 2019; Matsunaga et al., 2021). We have previously observed that direct mechanical stimulation-induced intracellular Ca^2+^ signals are almost completely abolished in human odontoblasts, which had Piezo1 channel knockdown using gene silencing with short hairpin RNA (shRNA), relative to the cells not subjected to shRNA-mediated knockdown of Piezo1 (Matsunaga et al., 2021). This previous result has indicated that contribution of Piezo2 channels on the mechanosensitive process in odontoblast is unlikely. Although the GsMTx-4 is known to suppress TRPC1 and TRPC6 channels in addition not only Piezo1 but also Piezo2 channels, TRPC1, TRPC4, TRPC5 and TRPC6 channel inhibitors did not inhibit any mechanical stimulation-induced Ca^2+^ entry in single odontoblasts (personal communication from YS). In the present results, compared to the inhibitory effect of the TRPA1 channel antagonist, inhibition of Piezo1 channels following systemic administration of GsMTx-4 almost completely abolished nociceptive behaviors after providing cold stimuli to exposed dentin. Therefore, Piezo1 channels are the most predominant mechanosensitive cation channels in odontoblasts, mediating sensory transduction for dentinal sensitivity caused by various stimuli, including cold stimuli. In addition, Piezo1 channels may act upstream of other mechanosensitive ion channels, including TRP and Piezo2 channels (personal communication from YS).

We further recorded dentinal sensitivity-related neuronal activities from the whole TG neurons when cold water was applied to the exposed dentin surface of the lower first molars. Although the whole TG contains cell bodies of primary sensory afferent innervating orofacial structures, such as dentin, pulp, periodontal tissues, masticatory muscles, jaw bones, facial skin, and oral mucosa. Mandibular nerves of trigeminal nerves innervate the site where the cold-noxious stimuli are applied in the present study. Primary sensory afferents of the mandibular nerve possess the slowly- and rapidly-adapting mechanoreceptors, nociceptors, and thermoreceptors. We could, however, observe increases in the spike frequencies of the cold stimulus-responded neurons during dentin cold stimuli. These activities were also sensitive to the intraperitoneal injection of the Piezo1 channel, P2X_3_ receptor, and PANX-1 inhibitors, indicating that together the results obtained in this study, the Piezo1–PANX-1–P2X_3_ axis in odontoblasts and neurons mediates sensory transduction in dentinal sensitivity.

**FIGURE 10:**
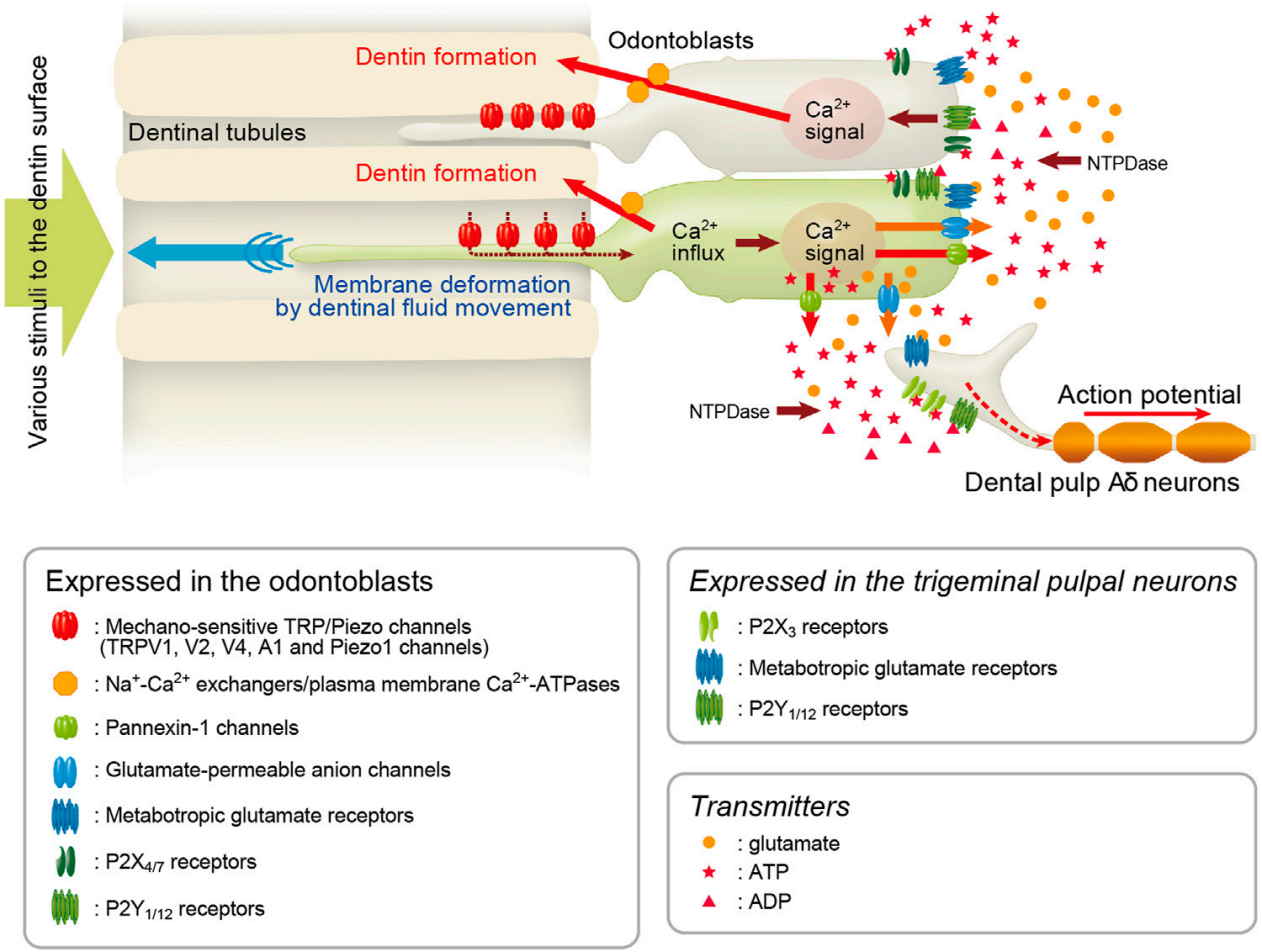
Odontoblast mechanosensory/hydrodynamic receptor model explains dentinal sensitivity Schematic representation of signal transduction pathway in odontoblast-neuron coupling in the generation of dentinal sensitivity, showing the “odontoblast mechanosensory (or hydrodynamic) receptor model.” Odontoblasts detect the cellular deformation by hydrodynamic force *via* dentinal fluid movements inside dentinal tubules using mechanosensitive Piezo1 channels, TRPVs, and TRPA1 channels. TRP/Piezo1-mediated intracellular Ca^2+^ signals elicit the release of ATP from ATP-permeable PANX-1 in odontoblasts. The released ATP acts as the predominant neurotransmitter for P2X_3_ receptors on pulpal Aδ neurons to drive the sensory transduction sequence underlying dentinal sensitivity. Additionally, glutamate released from glutamate-permeable anion channels by activation of mechanosensitive ion channels in odontoblasts activates metabotropic glutamate receptors (mGluRs) in neurons, establishing the odontoblast-neuron communication (Nishiyama et al., 2016) underlying dentinal sensitivity. During the mechanosensory transduction sequence in odontoblasts, increased intracellular Ca^2+^ levels following Ca^2+^ entry from TRP channels is extruded by Na^+^-Ca^2+^ exchangers (NCXs) and plasma membrane Ca^2+^-ATPase (PMCA) to drive reactionary (tertiary) dentinogenesis (Tsumura et al., 2010; Sato et al., 2013; Kimura et al., 2021). In addition to the sensory transduction mechanism, ADP hydrolyzed from ATP by nucleoside triphosphate diphosphohydrolases (NTPDases) and glutamate released from odontoblasts activate P2Y_1_ and P2Y_12_ receptors (Sato et al., 2015; Shibukawa et al., 2015), and mGluRs (Nishiyama et al., 2016), respectively, in the surrounding odontoblasts in an autocrine/paracrine manner, forming inter-odontoblast communication. Odontoblasts also functionally express P2X_4_ and P2X_7_ receptors (Lee et al., 2017; Shiozaki et al., 2017), which may contribute to paracrine signaling in odontoblast-odontoblast communication. The inter-odontoblast signal networks may also contribute to precise regulatory mechanisms in developmental/tertiary dentinogenesis in the dentin-pulp complex. In addition, exposure of the dentin surface to high pH stimuli (e.g., dental materials such as calcium hydroxide or mineral trioxide aggregate) induces intracellular Ca^2+^ mobilization *via* activation of TRPA1 channels and/or store-operated Ca^2+^ entry (SOCE) in odontoblasts (Shibukawa and Suzuki, 2003; Tsumura et al., 2013; Kimura et al., 2016, 2018). NCXs and PMCAs in odontoblasts are functionally coupled with high pH stimulation-induced Ca^2+^ mobilization, which is important for physiological and high-pH-induced dentinogenesis (Kimura et al., 2016, 2021). Our recent results also indicate “retrograde” communication between neurons and odontoblasts *via* neuropeptides, such as calcitonin gene-related peptide (CGRP), released from nerve terminals (i.e., axon reflex) during neurogenic inflammation in the dental pulp (Saito et al., 2022).

In conclusion (see Figure 10), our results show that activation of Piezo1 and TRPA1 channels and PANX-1 and P2X_3_ receptors, and odontoblasts themselves are necessary to generate dentinal sensitivity, *via* intercellular odontoblast-neuron signal communication. This indicates that odontoblasts act as mechanosensory receptor cells, and these mechanisms best describe the generation of dentinal sensitivity. The mechanism has been proposed as the “odontoblast hydrodynamic receptor model” (Shibukawa et al., 2015); however, the “*odontoblast mechanosensory receptor model*” should be suitable to describe the generation mechanisms in a more inclusive sense. It has been well described that dentinal fluid movement inside dentinal tubules (Gysi, 1900; Bränström, 1963; Andrew and Matthews, 2000; Charoenlarp et al., 2007; Vongsavan and Matthews, 2007) by various stimuli to the dentin surface results in the cellular deformation of odontoblasts as mechanical stimulation (Lin et al., 2011), however, we did not observe any effects of the fluid movement on the painful behavior, except the result showing reduction of nociceptive scores by the bonding agent in the present study. Thus, our immediate interest is to clarify the relationship between dentinal fluid movements and the development of dentinal sensitivity *via* odontoblasts as mechanosensory receptor cells. The present findings provide an explicit and robust understanding of dentin hypersensitivity and new strategies for treating and managing this condition.

## Data Availability

The original contributions presented in the study are included in the article/Supplementary Material, further inquiries can be directed to the corresponding author.
